# A preliminary study of telemedicine for patients with hepatic glycogen storage disease and their healthcare providers: from bedside to home site monitoring

**DOI:** 10.1007/s10545-018-0167-2

**Published:** 2018-03-29

**Authors:** Irene J. Hoogeveen, Fabian Peeks, Foekje de Boer, Charlotte M. A. Lubout, Tom J. de Koning, Sebastiaan te Boekhorst, Robert-Jan Zandvoort, Rob Burghard, Francjan J. van Spronsen, Terry G. J. Derks

**Affiliations:** 1 Section of Metabolic Diseases, Beatrix Children’s Hospital University Medical Center Groningen, University of Groningen, PO Box 30 001, 9700 RB Groningen, The Netherlands; 2 Patient Connect B.V., Bilthoven, The Netherlands; 3 Pi IQ, Groningen, the Netherlands; 4 enerGQcare B.V., Groningen, The Netherlands

**Keywords:** Hepatic glycogen storage diseases, Telemedicine, Monitoring, Mobile application, Dietary management, Rare diseases

## Abstract

**Background:**

The purpose of this project was to develop a telemedicine platform that supports home site monitoring and integrates biochemical, physiological, and dietary parameters for individual patients with hepatic glycogen storage disease (GSD).

**Methods and results:**

The GSD communication platform (GCP) was designed with input from software developers, GSD patients, researchers, and healthcare providers. In phase 1, prototyping and software design of the GCP has occurred. The GCP was composed of a GSD App for patients and a GSD clinical dashboard for healthcare providers. In phase 2, the GCP was tested by retrospective patient data entry. The following software functionalities were included (a) dietary registration and prescription module, (b) emergency protocol module, and (c) data import functions for continuous glucose monitor devices and activity wearables. In phase 3, the GSD App was implemented in a pilot study of eight patients with GSD Ia (*n* = 3), GSD IIIa (*n* = 1), and GSD IX (*n* = 4). Usability was measured by the system usability scale (SUS). The mean SUS score was 64/100 [range: 38–93].

**Conclusions:**

This report describes the design, development, and validation process of a telemedicine platform for patients with hepatic GSD. The GCP can facilitate home site monitoring and data exchange between patients with hepatic GSD and healthcare providers under varying circumstances. In the future, the GCP may support cross-border healthcare, second opinion processes and clinical trials, and could possibly also be adapted for other diseases for which a medical diet is the cornerstone.

## Introduction

Hepatic glycogen storage diseases (GSDs) are a group of inborn errors of carbohydrate metabolism, for which a strict diet is the cornerstone of management. Patients with hepatic GSD display perturbed glucose homeostasis due to a deficiency of a functional enzyme or transporter in glycogen synthesis, glycogenolysis, and/or gluconeogenesis. This generally leads to fasting intolerance, failure to thrive and hepatomegaly, and is biochemically associated with (non)ketotic hypoglycemia, (fasting or postprandial) hyperlactacidemia, increased liver enzymes and hyperlipidemia (Walter et al 2016). The aim of dietary management is to maintain euglycemia, to suppress secondary metabolic derangements and to prevent long-term complications. The natural histories of several subtypes of hepatic GSD were reported by international cohort studies (Rake et al 2002a; Sentner et al 2016), review articles (Bali et al 2006; Dagli & Weinstein 2009; Goldstein et al 2011; Dagli et al, 2010; Magoulas & El-Hattab 2013), and guidelines (Rake et al 2002b; Visser et al 2002; Kishnani et al 2010; Kishnani et al 2014).

In line with the observations that strict diabetes management prevents long-term complications, observational cohort studies of GSD I patients have emphasized the importance of good metabolic control for the prevention of liver adenomas (Wang et al 2011), nephropathy (Wolfsdorf et al 1997; Martens et al 2009; Melis et al 2015; Okechuku et al 2017) and bone disease (Minarich et al 2012; Melis et al 2014). For GSD IIIa patients, an association between overtreatment with carbohydrates and hypertrophic cardiomyopathy was suggested by several case-reports (Dagli et al 2009; Valayannopoulos et al 2011; Sentner et al 2012). In addition, a case report on two patients with GSD IXa presented improvement in liver cirrhosis on ultrasound after improving metabolic control (Tsilianidis et al 2013).

Continuous glucose monitoring (CGM) systems have been developed to facilitate glucose monitoring. Originally, CGM systems were developed for patients with diabetes mellitus (DM) (Juvenile Diabetes Research Foundation Continuous Glucose Monitoring Study Group 2008), but the application of CGM in GSD patients has also been reported by several groups (Hershkovitz et al 2001; Maran et al 2004; White & Jones 2011; Kasapkara et al 2014). The introduction of CGM systems has increased opportunities for home site monitoring. Nevertheless, day-to-day healthcare for the GSD patients is still challenging of which suboptimal metabolic control is only one of many reasons (as summarized in Table 1).

**Table 1:** Challenges in current healthcare for individual patients with hepatic GSD

1.	Suboptimal metabolic control (due to under- or overtreatment with carbohydrates) still occurs, associated with co-morbidity and long-term complications.
2.	There is a gap of knowledge between clinical guidelines and management in daily practice.
3.	There is large heterogeneity between individual patients with identical GSD subtypes and genotypes
4.	There is a discrepancy between prescribed diets and actual used diets.
5.	Clinical parameters are mostly measured in the hospital on relatively random moments.
6.	Traditional biomarkers are suboptimal and biochemically distant from the primary metabolic block.
7.	Patients with rare diseases usually do not live close to so-called centers of expertise, which challenges ‘shared care models’.

Telemedicine (TM) has emerged rapidly as a novel tool to deliver healthcare tailored to the individual patient’s needs (Moore 1999; Steinhubl et al 2015; Heintzman 2016). Besides this, TM seems promising to provide cross-border healthcare for patients with rare diseases (Saliba et al 2012). Surprisingly, to date, there is only one publication on a mobile application for dietary management of patients with inborn errors of metabolism (Ho et al 2016).

To support home site monitoring and to integrate biochemical, physiological, and dietary parameters, we have designed, validated, and implemented a TM platform for patients with hepatic GSD. This TM platform, now further referred to as GSD communication platform (GCP), consists of the GSD application (GSD App) for patients and their caregivers and the GSD clinical dashboard for healthcare providers.

## Methods

The GCP was intended to *support* rather than replace current healthcare as provided by our center of expertise for hepatic GSD and recommendations were only generated after approval by a healthcare provider. The usability pilot study has been carried out in accordance with ethical and legal guidelines of the University Medical Center Groningen (Medical Ethical Committee, 2016/466) and The Code of Ethics of the World Medical Association (Declaration of Helsinki). Technical documentation and user manuals of the GCP were prepared for the validation of the GCP as a class I medical device, as described in the Medical Device Directive 93/42/EEC. The creation of the technical documentation was supported by DRS consultancy (http://drs.nu/en_US/) and subsequently reviewed by the quality consultant medical devices of the University Medical Center Groningen, in preparation of notifying the GCP to the national Inspectorate of Health for a Conformité Européenne (CE) mark.

For the software development and validation process of the GCP, three phases were constructed.

**Fig. 1:**
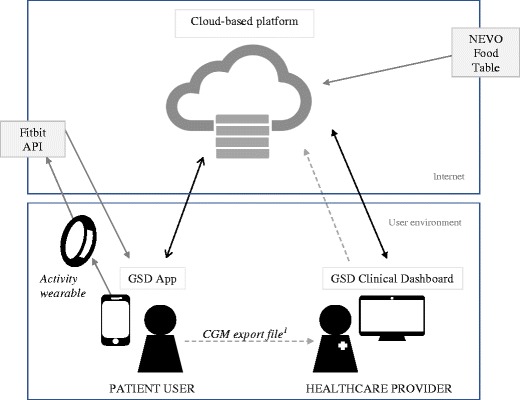
Architecture of the GSD communication platform. Legend: ^1^, no API was available for the CGM data, these data were manually imported to the GSD clinical dashboard by the healthcare provider. API, application programming interface; CGM, continuous glucose monitoring; GSD, glycogen storage disease

- Phase I — Prototyping and software design:
  The GCP was designed during monthly meetings with input from software developers, (parents of) GSD patients, researchers, and healthcare providers from June 2014 till present. The GCP was composed of two web applications; the GSD App for patients and their caregivers and the GSD clinical dashboard for healthcare providers. The GSD App was developed in Dutch, the native language for most of our patients, whereas the GSD clinical dashboard was developed in English. Figure 1 presents the detailed architecture of the GCP.
  - Intended uses — The intended use of the GSD App is to allow individuals with hepatic GSD to support their dietary management provided by healthcare providers, under normal circumstances and intercurrent illness, by monitoring and sharing home site collected data with healthcare providers. The intended use of the GSD clinical dashboard is to integrate data collected by hepatic GSD patients, either at home or during a hospital admission, and to provide subsequent GSD dietary management advice for normal circumstances, intercurrent illness, and those situations where the emergency protocol is applicable.
  - Data security and management — For both applications, the Hypertext Transfer Protocol Secure (HTTPS) and the Open Web Application Security Project Top Ten awareness document (https://www.owasp.org/) was adopted to secure integrity and privacy of online communication within the GCP. Access to the applications required a username and password. Passwords were stored with a salted and one-way encryption method to protect from accidental or unlawful loss. Software test plans and complaints management procedures were set up for post-market evaluation. The GCP was hosted on Microsoft Azure (refer to Azure subscription agreement: https://azure.microsoft.com/en-us/support/legal/subscription-agreement/). For the GSD App development, the open source frameworks AngularJS, JavaScript jQuery, and Bootstrap were chosen. A non-commercial license from Highcharts (https://www.highcharts.com) was used to display the graphs in the GSD clinical dashboard.
- Phase II — Software development and retrospective clinical data entry:
  Features and functionality were reviewed and issues on usability were managed, processed, and documented with the use of a Jira issue tracker from Atlassian® by software developers, researchers, and healthcare providers.
  The following software functionalities were included:
  - Dietary registration and prescription module — The Dutch Food Composition Table (NEVO table) (National Institute for Public Health and the Environment 2012) was used for the development of the dietary registration module in the GSD App and the prescription module for dieticians in the GSD clinical dashboard, respectively. The NEVO table contains information on macro- and micronutrients content and total kilocalories of all food items frequently eaten by the Dutch population. The NEVO table also includes data on the medical formulas, dietary supplements, maltodextrin products, and uncooked cornstarch, such as Glycosade®.
  - Emergency protocol module — The local hospital emergency protocol guideline was used as a template for the emergency protocol module in the GSD clinical dashboard. The emergency protocol module was designed in such a way that it could automatically generate an emergency letter with the use of the patient’s GSD type and actual body weight. After generation, the emergency letter was shared with the corresponding patient in the GSD App.
  - Data import functions — In the GSD clinical dashboard, an import function for CGM data (Dexcom G4/G5 CGM system, Dexcom Inc., San Diego, CA) was created. An Application Programming Interface (API) was acquired by Fitbit, Inc. to import data from the activity wearable in the GSD App.
  - Retrospective data entry — Data from written food and clinical measurement diaries were retrospectively collected in the GCP by the researchers to test the usability and correctness of the GCP functionalities. These data were from patients who visited the University Medical Center Groningen GSD center of expertise between March and October 2016 and who gave written informed consent for the use of their data collected during their visit.
- Phase III — Implementation and pilot study prospective clinical data entry:
  - Subjects and pilot study design — Between March and July 2017, data were prospectively collected in the GCP by selected GSD patients visiting our center. Patients were introduced to the GSD App by the treating physician (TGJD) and researcher (IJH). All subjects received an up-to-date manual for the use of the GSD App. Data exchange was requested by the healthcare provider to allow individual data integration in the GCP and critical follow-up after dietary changes. Subjects were asked to use the GSD App for home site monitoring before, during, and/or after a clinic visit, according to the purpose of their visit. Furthermore, subjects could temporarily use an activity wearable with a heart rate function (Fitbit Charge HR™). A CGM system by Dexcom was used only when needed for regular care. Data from the CGM were retrospectively imported in the GSD clinical dashboard by the healthcare providers. Results of data integration in the GSD clinical dashboard (i.e., updated dietary plans and emergency protocols) were discussed with the subjects/patients and their parents during the outpatient clinic visit and/or via a telephone or videoconference consultation following the (outpatient) clinic visit.
  - Survey methods — User feedback was documented in a structured logbook and usability issues from different users (patients, caregivers, and healthcare providers) were uploaded in the Jira issue tracker for further software improvements. Subjects and/or caregivers were asked to give feedback on the GSD App via an open feedback form on paper or electronically via a SurveyMonkey questionnaire and to fill in the system usability scale (SUS) (Brooke 1996). An adjective scale was used for the interpretation of individual SUS scores (Bangor et al 2009).

## Results

### Phase I: prototyping and software design

**Fig. 2:**
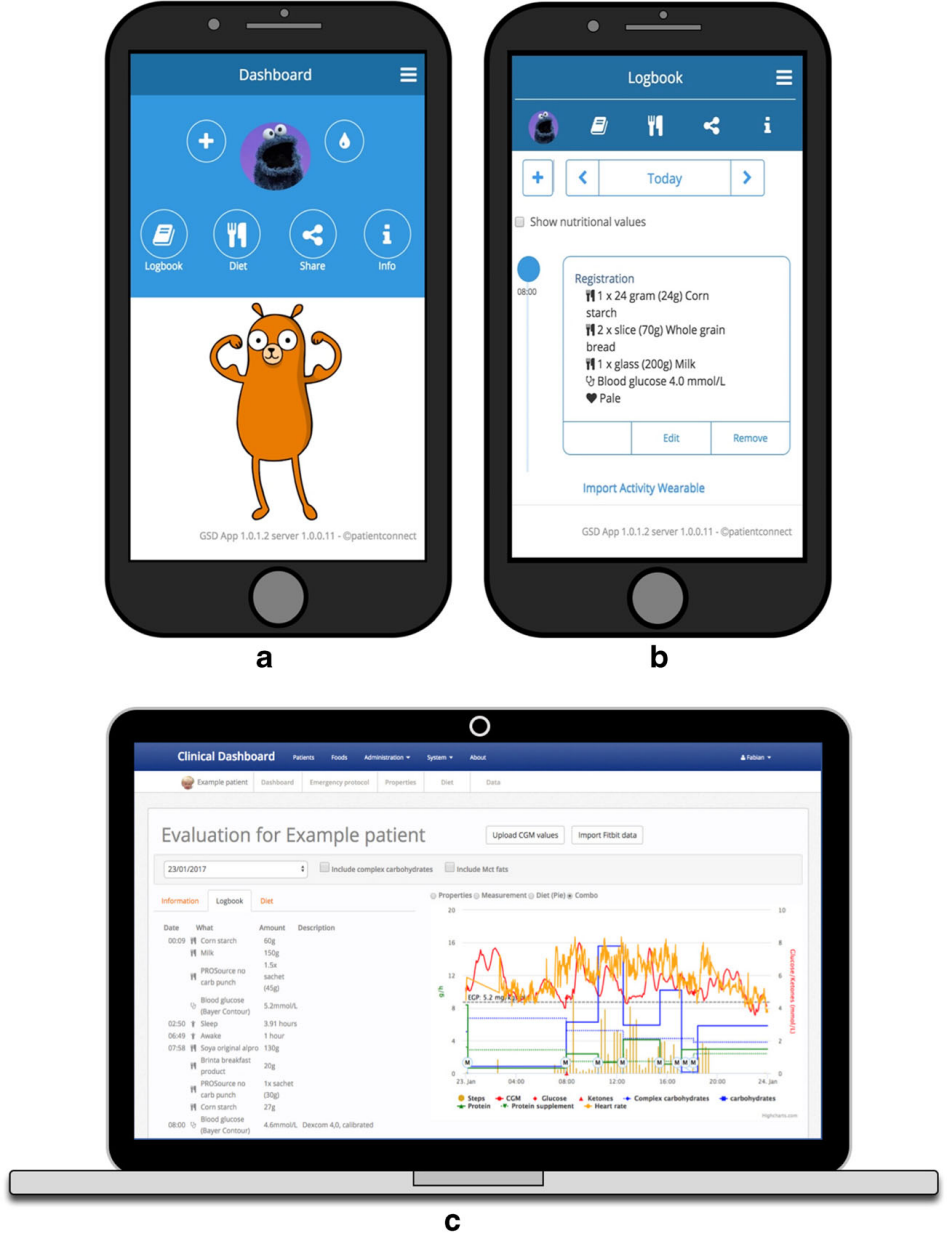
**Screenshots from the GSD App and the GSD clinical dashboard: a) the home screen, b) the logbook, c) evaluation display in the GSD clinical dashboard**. Legend: a) the cutlery button presents the prescribed diets; the share button displays the users and the shared data periods. The emergency protocol and personal emergency phone numbers are accessible via the information button. b) the logbook gives a clear overview of all registered meals over the day in a chronological manner. Macronutrient totals can be displayed per food item, per meal, and/or per day. Besides dietary registration, the GSD App allows patients to enter their blood glucose/ketones measurements, physical activities, and symptoms. c) the combo graph in the GSD clinical dashboard presents all individual data in one interactive graph. Data sets can be added or deleted from the graph

Figure 2 displays screenshots of the GSD App and the GSD clinical dashboard. The GSD App can be accessed by going to https://app.gsdapp.nl in the internet browser on a mobile phone. The GSD clinical dashboard can be accessed via a desktop browser: https://clinicaldashboard.gsdapp.nl.

### Phase II: software development and retrospective clinical data entry

Access to individual patient registered data in the GSD App was restricted to the patient user and their caretakers. Data share requests by healthcare providers via the GSD clinical dashboard (for instance, for the preparation of an outpatient clinic visit) were only answered by and with the permission of (the caretakers of) the patient.

#### Data import and visualization

The combo graph in the GSD clinical dashboard displayed combined data of the patient from the requested period in one interactive graph (see Fig. 2c). The GSD clinical dashboard also displayed a histogram and a pie chart to provide more insight into the differences in macronutrient distributions and total calorie intake between prescribed and registered diets.

#### Dietary prescription module

The implementation of the NEVO table supported dietary registration, prescription, and macronutrient calculations. An extra function was added for the entry of drip-feeding to simplify the determination of the rate of drip-feeding in milliliters per hour and the macronutrient intake.

#### Retrospective data entry

Table 2 summarizes retrospective data on dietary management and CGM profiles from five patients with GSD Ia (*n* = 3), GSD IIIa (*n* = 1), and GSD IX (*n* = 1). Improvements for dietary registration in the GSD App and data visuals in the GSD clinical dashboard were implemented. Eventually, retrospective data entry led to the development of the GSD App and GSD clinical dashboard versions for the usability pilot study in phase III.

**Table 2:** Patient characteristics for phase II and III data collection in the GCP

Pt nr.	Age^a^	GSD type	Sex	Purpose	Logbook entry^b^	CGM import	Activity import^c^	Dietary advice	
Phase II: retrospective clinical data entry									
R01	12	Ia	F	Nocturnal Glycosade® versus CNGDF	✓	✓	✓	✓	
R02	31	Ia	M	Regular outpatient clinic visit for metabolic monitoring	✓	✓	✓	✓	
R03	36	IIIa	F	Monitoring introduction of MCT	✓	✓	✓	✓	
R04	0	IX	M	Metabolic monitoring during breastfeeding on demand	**–**	✓	**–**	**–**	
R05	19	Ia	M	Metabolic monitoring	✓	✓	**–**	✓	
Phase III: pilot study prospective clinical data entry									SUS score (1–100)
P01	13	Ia	F	^d^Nocturnal Glycosade® versus CNGDF	✓	✓	**–**	✓	38
P02	0	IX	M	^d^Regular outpatient clinic visit for metabolic monitoring	✓	✓	**–**	✓	*no response*
P03	31	Ia	F	Introduction of Glycosade® as late night drink	✓	✓	**–**	✓	55
P04	1	IX	M	Regular outpatient clinic visit for metabolic monitoring	✓	✓	**–**	✓	93
P05	50	IX	M	Monitor of sport regimen	✓	**–**	✓	–	63
P06	4	IIIa	F	Regular outpatient clinic visit for metabolic monitoring	✓	**–**	**–**	✓	75
P07	20	Ia	M	^d^Regular outpatient clinic visit for metabolic monitoring	✓	✓	**–**	✓	45
P08	5	IXc	M	Regular outpatient clinic visit for metabolic monitoring	✓	**–**	**–**	✓	80

*CGM* continuous glucose monitoring, *CNGDF* continuous nocturnal gastric drip feeding, *MCT* medium chain triglycerides, *SUS* system usability scale

^a^Age of the patient during data period entered in the GSD App

^b^Logbook entry included dietary registration and measurements

^c^Import of activity wearable data by Fitbit

^d^This patient was also included in the retrospective clinical data entry

### Phase III: implementation and pilot study prospective clinical entry

#### Subjects

The GSD App was prospectively used by eight patients with GSD type Ia (*n* = 3), IIIa (*n* = 1), and IX (*n* = 4). Table 2 presents an overview of the patients’ characteristics. Three patients participated in both the retrospective clinical data entry and the pilot study. The GSD App was used by both GSD patients (*n* = 3) and caregivers (*n* = 5). Patient #P01 used the GSD App together with her parents as a first step toward autonomy.

#### Pilot study outcome

Table 2 displays the different purposes for which the GSD App was used. In phase III (P02, P04-P08), data were collected in the GSD App outside the hospital environment. The distance to our hospital ranged between 158 and 397 km, emphasizing the potential in home site monitoring. Only one patient used the activity wearable (Fitbit Charge HR™) to monitor heart rate and activity as an extra physiological marker for the assessment of his sport regimen.

#### Feedback from users

Feedback on the GSD App could be collected for 7/8 GSD App users (see Table 2), unfortunately #P02 has not returned written feedback, despite reminders. The mean SUS score was 64/100 [range: 38–93]. Two subjects scored the GSD App usability with ‘excellent’ on the adjective scale for interpretation of individual SUS scores. The mean SUS was higher among parents with younger children (*n* = 3, mean SUS 83) compared to adult patients or parents with older children (≥12 years of age) (*n* = 4, mean SUS 50). Patients reported in the open feedback form that it was easy to have their dietary prescription at hand and that the GSD App worked more efficiently compared to the paper food diaries. Points for improvements were the search strategy for the right food items and the feedback from healthcare providers on registered data.

## Discussion

This report describes the design, development, and validation process of a TM platform for patients with hepatic GSD. The GCP can facilitate home site monitoring and data exchange between patients with hepatic GSD and healthcare providers under varying circumstances. Rather than a *study* or a *project*, the development and maintenance of a TM platform for medical use is a continuous *process* responding to individual needs of patients and healthcare providers, technological and societal advancements, and the changing (international) legislation, as discussed below.

The GCP has been developed as a supportive tool for GSD patients and healthcare providers in our center of expertise. The GSD App allows patients to easily collect data at home as a preparation for an outpatient clinic visit, for the monitoring of in-hospital dietary interventions, and for the evaluation of metabolic control at home and during physical activity. In addition, the GSD App gives patients access to their individual dietary management plan and emergency letter. Based on the collected SUS scores in the pilot study, it can be suggested that parents of GSD patients are in more need of a home site monitoring tool like the GSD App to manage everyday care for their child. The GSD clinical dashboard for healthcare providers facilitates diet prescription and emergency letter generation. Furthermore, the GSD clinical dashboard offers healthcare providers the possibility to analyze home site collected data from the individual patient. For several hepatic GSD subtypes there are international guidelines (Rake et al 2002b; Visser et al 2002; Kishnani et al 2010; Kishnani et al 2014), but local and national circumstances need to be considered when the GCP would be implemented in other centers of expertise.

The connection of measure devices with the patient can be defined as the Internet of things (IoT) in healthcare (Dimitrov 2016). IoT allows real-time and home site collected data sharing and integration, and eventually a better response to individual data. In general, data ownership within IoT is still in the gray area, because of the absence of transparent data regulation (Dinesen et al 2016; Kostkova et al 2016). We created different interactivities between the GCP and other technologies, such as the CGM systems and activity wearables. During the pilot study, data visuals within the GCP were only accessible for healthcare providers in the GSD clinical dashboard. An important point of feedback from the patients and their parents was the lack of interactivity of home site collected data and the visibility of these data in the GSD App for the patients themselves.

The revolution of the internet has generated increasing interest for home site or remote monitoring in healthcare that can overcome many of the issues listed in Table 1. The Dutch Federation of Medical Specialists published the vision document Medical Specialist 2025 that lists four essential requirements for future healthcare: (a) a holistic approach by the healthcare provider for each individual, unique patient, (b) digital developments to support network medicine, (c) focus on health, behavior, and functional maintenance to prevent disease, and (d) implementation of big data analysis, wearables, and home diagnostics in self-management of chronic diseases. More recently, Augustine and co-workers reported a novel healthcare model for patients with rare diseases that focusses on TM, integration of care and research, and improving patient–clinician–researcher collaborations (Augustine et al 2017). In line with the so-called care continuum model, continuous maintenance and improvement of the GCP will occur in collaboration with the patients and their caregivers. Currently, there is only one report on a mobile application for dietary management of patients with inborn errors of metabolism (Ho et al 2016). Our project may serve as an example to design, validate, and implement a unique TM platform for and together with patients with rare diseases.

Some limitations need to be addressed. First, we performed a pilot study with a small number of patients only focusing on the usability and functionalities of the GSD App. Future research must focus on the effect of the GCP intervention on metabolic control, quality of life, and cost-effectiveness. Usability of the GSD clinical dashboard for healthcare providers should also be studied. Second, new versions of the GSD App have been released in between the collection of SUS scores. Continuous improvement of the GSD App based on patient’s feedback was possible due to close collaboration with the patient population and software developers, but therefore SUS score interpretation should be done with caution. Third, for the development of the GCP, the team collaborated especially with parents of GSD patients, hence the needs of (young) patients may have been underestimated.

Currently, the European Medical Device Directive 93/42/EEG is still the legal fundament guiding CE mark procedures of medical software, whereas ISO 14155:2011 addresses the good clinical practice of clinical investigations with humans to assess the safety and performance of medical devices. Since the start of our project, however, in our rapidly changing technological society, new European legislation has been approved regarding medical devices (i.e., the Medical Device Regulation MDR 2017/745) and data privacy (i.e., the General Data Protection Regulation). Hence, technical, societal, medical, and legal developments are continuously influencing the process, demonstrating the complex dependency of different stakeholders to support healthcare for our patients and their caregivers.

Future perspectives and applications of the GCP are numerous. The platform could facilitate cross-border healthcare, support second opinion processes, and provide support during clinical trials. In theory, the GCP could be adapted for patients with congenital hyperinsulinism and ketogenic diets (i.e., other rare conditions in which glucose homeostasis is perturbed) and inherited metabolic diseases for which a medical diet is the cornerstone. For these purposes, multiple translations and integration of the GCP with national food databases and electronic patient file systems will be crucial. The — in 2016 formally recognized — European Reference Network for Rare Hereditary Metabolic Disorders (MetabERN) could support the generation and (financial) maintenance of such TM projects (Raposo 2016; Héon-Klin 2017), or alternatively, patient organizations.

## Abbreviations

API: Application programming interface
CE: Conformité Européenne
CGM: Continuous glucose monitoring
GCP: GSD communication platform
GSD: Glycogen storage disease
DM: Diabetes mellitus
IoT: Internet of things
MetabERN: European Reference Network for Rare Hereditary Metabolic Disorders
SUS: System usability scale
TM: Telemedicine
